# Microencapsulation of Coffee Pulp Extract (*Coffea arabica*) with Maltodextrin and Whey Protein Isolate: Physicochemical Characterization, Antioxidant Capacity, and Antimicrobial Activity

**DOI:** 10.3390/molecules31213927

**Published:** 2026-11-01

**Authors:** Maria Fernanda Zamora-Montealegre, Dalila Canizales-Rodríguez, Saúl Ruiz-Cruz, Esteban Largo-Avila, Jesús Fernando Ayala-Zavala, Melvin Roberto Tapia-Rodríguez, Carmen Lizette Del-Toro-Sánchez, Irela Santos-Sauceda, Silvia Elena Burruel-Ibarra, Teresita de Jesús Moreno-Pérez, Alba Mery Garzón-García

**Affiliations:** 1 Maestría en Biotecnología, Universidad del Valle, Cali 760032, Valle del Cauca, Colombia; maria.montealegre@correounivalle.edu.co; 2 Departamento de Ciencias Químico Biológicas, Universidad de Sonora, Hermosillo 83000, Sonora, Mexico; dalila.canizales@unison.mx; 3 Departamento de Investigación y Posgrado en Alimentos, Universidad de Sonora, Hermosillo 83000, Sonora, Mexico; saul.ruizcruz@unison.mx (S.R.-C.); carmen.deltoro@unison.mx (C.L.D.-T.-S.); 4 Sistema de Regionalización, Universidad del Valle, Caicedonia 762540, Valle del Cauca, Colombia; 5 Centro de Investigación en Alimentación y Desarrollo, A.C. (CIAD), Hermosillo 83304, Sonora, Mexico; jayala@ciad.mx; 6 Departamento de Biotecnología y Ciencias Alimentarias, Instituto Tecnológico de Sonora, Ciudad Obregón 85000, Sonora, Mexico; melvin.tapia14987@potros.itson.edu.mx; 7 Departamento de Investigación en Polímeros y Materiales, Universidad de Sonora, Hermosillo 83000, Sonora, Mexico; irela.santos@unison.mx (I.S.-S.); silvia.burruel@unison.mx (S.E.B.-I.); teresita.moreno@unison.mx (T.d.J.M.-P.)

**Keywords:** coffee by-product, aqueous extract, encapsulation, freeze-drying, biopolymers, biological activity, structural characterization

## Abstract

Coffee production generates large volumes of by-products rich in bioactive compounds with biological potential, such as coffee pulp. This study evaluated microencapsulation by freeze-drying of aqueous coffee pulp extract using maltodextrin, whey protein isolate, and a 1:1 combination of both as wall materials. Lyophilization yields ranged from 78.99% to 81.49%. SEM analysis revealed a lamellar morphology characteristic of freeze-dried powders in all formulations. FT-IR suggested molecular interactions between wall materials and extract compounds, with Amide I and II bands attenuated in EMP. TGA showed a distinctive degradation event at 513 °C in EMP, which was absent in the controls without extract. Encapsulation efficiency exceeded 94% in all formulations, with EPR and EMP reaching values close to 99%. Among the formulations, EPR showed the highest antioxidant capacity in ABTS and FRAP assays. All formulations showed inhibitory and bactericidal activity against *Escherichia coli* and *Staphylococcus aureus*, with EMD showing the lowest minimum bactericidal concentration against *E. coli* (1 mg/mL). These results indicate that each wall material could confer different properties to the microparticles, supporting freeze-drying microencapsulation as a strategy for the valorization of bioactive compounds from coffee pulp.

## 1. Introduction

Coffee cultivation is one of the most relevant agricultural activities worldwide, with a profound impact on the economic, social, and cultural sectors of producing regions [1]. Before commercialization, coffee undergoes several processing stages during which the pulp, mucilage, and husk are separated from the bean. Since only the bean is used to obtain the final product, approximately 90% of the coffee cherry is discarded [2,3]. This generates large volumes of residues that, when not properly managed, represent an environmental risk and a loss of high value-added compounds [4]. Currently, these residues are primarily used for composting, without maximizing their potential [5,6].

Coffee pulp is an important source of bioactive compounds, including polyphenols such as hydroxycinnamic acids, flavanols, and anthocyanidins, as well as chlorogenic acids, gallic acid, and 5-caffeoylquinic acid [7,8]. These compounds have demonstrated antioxidant, anti-inflammatory, and antimicrobial properties against Gram-positive bacteria such as *Staphylococcus aureus* and Gram-negative bacteria such as *Escherichia coli* [9,10]. However, their direct application is limited by their marked instability against environmental factors such as pH, light, and temperature, which promote their oxidation and degradation [11,12].

Microencapsulation has emerged as an effective strategy to overcome these limitations, as it protects bioactive compounds from environmental degradation, improves their thermal stability and bioavailability and facilitates their incorporation into food, cosmetic, or pharmaceutical formulations [13,14,15]. Among the available encapsulation methods, freeze-drying stands out because it removes water by sublimation at low temperatures, minimizing thermal and oxidative degradation of sensitive metabolites, unlike spray drying, which involves exposure to high temperatures [16,17]. In this regard, the choice of wall materials is fundamental to the effectiveness of the encapsulation process. Maltodextrin and whey protein isolate are widely used in the microencapsulation of bioactive compounds due to their biocompatibility, solubility, and protective capacity [18,19,20,21]. Maltodextrin forms stable dry matrices, although with limited protection against oxidation, while whey protein isolate provides excellent emulsifying properties and the ability to interact with phenolic compounds through electrostatic interactions, improving the retention of active compounds [18,19,20,21]. Their combined use has shown promising results in terms of encapsulation efficiency and final product stability [22,23].

Several studies have addressed the microencapsulation of phenolic compounds from coffee by-products, using different biomass sources, extraction methods, wall materials, and drying techniques. Ballesteros et al. [24] encapsulated an extract obtained by autohydrolysis of spent coffee grounds with maltodextrin, gum arabic, and their mixture, and found that freeze-drying with maltodextrin provided the highest retention of phenolic compounds and flavonoids. Also working with spent coffee grounds, Araújo et al. [25] encapsulated an aqueous extract by spray-drying and freeze-drying with egg albumin, maltodextrin, gum arabic, and their mixtures, and observed that albumin showed the lowest retention of bioactive compounds, whereas the combination of gum arabic and maltodextrin provided the best preservation. In coffee husk, Silva et al. [15] encapsulated a hydroethanolic extract by spray-drying with maltodextrin, whey protein concentrate, and their blends, and found that formulations containing the protein favored the retention of polyphenols and increased their antioxidant capacity after simulated intestinal digestion, while Barbosa et al. [26] encapsulated an aqueous extract of coffee silverskin by spray-drying with whey protein concentrate and skimmed milk powder, preserving its phenolic content and antioxidant activity. For coffee pulp, Vázquez-Núñez et al. [27] encapsulated an acetone extract by spray-drying and found that whey protein was the most effective wall material for retaining procyanidins, whereas maltodextrin was the least effective.

Overall, these studies have mainly evaluated the retention of phenolic compounds, antioxidant capacity, and release during digestion, but, to our knowledge, not the antimicrobial activity of the microparticles, and only one of them has addressed coffee pulp. In addition, few studies have compared single- and binary-wall materials under the same processing conditions while including extract-free controls to distinguish the contribution of the wall materials from that of the extract. The Castillo^®^ cultivar was selected because it is one of the main rust-resistant varieties developed in Colombia, which currently occupies most of the country’s coffee-growing area [28]; therefore, its pulp represents a by-product generated in large volumes in the producing regions of the country. In this context, the present study evaluated the microencapsulation by freeze-drying of aqueous coffee pulp extract from the Castillo^®^ cultivar using maltodextrin (EMD), whey protein isolate (EPR), and a 1:1 combination of both (EMP) as wall materials, characterizing the obtained microparticles in terms of physicochemical properties, surface morphology, molecular interactions, thermal behavior, encapsulation efficiency, total phenolic and flavonoid contents, antioxidant capacity, and antimicrobial activity.

## 2. Results

Three microencapsulation formulations were evaluated: maltodextrin as wall material with coffee pulp extract as core (EMD), whey protein isolate with coffee pulp extract (EPR), and a 1:1 combination of maltodextrin and whey protein isolate with coffee pulp extract (EMP). Control formulations were prepared by replacing the extract with distilled water: maltodextrin (AMD), whey protein isolate (APR), and a 1:1 combination of both materials (AMP). Dried, ground, and sieved coffee pulp (ES), pure maltodextrin (MD), and pure whey protein isolate (PR) were also analyzed. ES was included as a reference of the raw material from which the extract was obtained, while the control formulations were used to identify the contribution of the wall materials to each analysis, so that the effect of the extract in the encapsulated formulations could be evaluated by comparing each formulation with its respective control.

### 2.1. Lyophilization Yield

Lyophilization yield was calculated considering the solids contributed by the coffee pulp extract (2.7 °Brix) and the wall materials. Extract-containing formulations showed similar yields among themselves, with values of 81.17% (EMD), 81.49% (EMP), and 78.99% (EPR), while control formulations AMD and APR showed higher yields of 86.55% and 84.41%, respectively. The yield of AMP (68.17%) is not representative, since part of the powder was accidentally lost during grinding, before weighing. The remaining powder, obtained from the same freeze-dried batch, was used for all subsequent analyses; therefore, this loss affected only the yield calculation and not the other analyses.

### 2.2. Characterization of Microparticles

#### 2.2.1. Moisture Content, Water Activity, and Color

Moisture content, water activity, and color analysis results are presented in Table 1. ES showed the highest moisture content (9.97 ± 0.03%), followed by control formulations and extract-containing formulations, among which EMP showed the lowest value (1.88 ± 0.04%), significantly lower than EMD (4.40 ± 0.20%) and EPR (3.62 ± 0.25%). Regarding water activity, AMD showed the highest value (0.569 ± 0.004), while EMP showed the lowest among all formulations (0.321 ± 0.003).

In the color analysis, all formulations showed statistically significant differences among themselves in all evaluated parameters (*p* < 0.05); however, numerical differences between some formulations were small. ES showed the lowest lightness (L* = 40.189 ± 0.002), being visually the darkest powder, with the highest color saturation (C* = 29.363 ± 0.006) and greatest tendency toward yellow-reddish hues (a* = 13.646 ± 0.004; b* = 26.000 ± 0.009). Extract containing formulations (EMD, EPR, and EMP) and controls (AMD, APR, and AMP) showed statistically different but numerically close values across all CIELab and CIELCh parameters, with differences practically imperceptible to the naked eye. EMD, EPR, and EMP showed high lightness values (83.003–84.206) with a tendency toward yellow-reddish hues, while controls showed the highest L* values (92.729–94.633), reflecting their lighter appearance in the absence of extract and a tendency toward pure yellow.

The total color difference (∆E), calculated using ES as reference, confirmed that encapsulation notably modified color across all formulations, with values between 44.802 (EPR) and 46.052 (EMD), well above the visual perception threshold (∆E > 3). The hue angle (h°) showed that ES was in a yellow-reddish color region (62.307°), encapsulated formulations showed intermediate values (78.323–78.756°), and controls approached more neutral hues (90.814–92.055°).

#### 2.2.2. Scanning Electron Microscopy (SEM)

**Figure 1:**
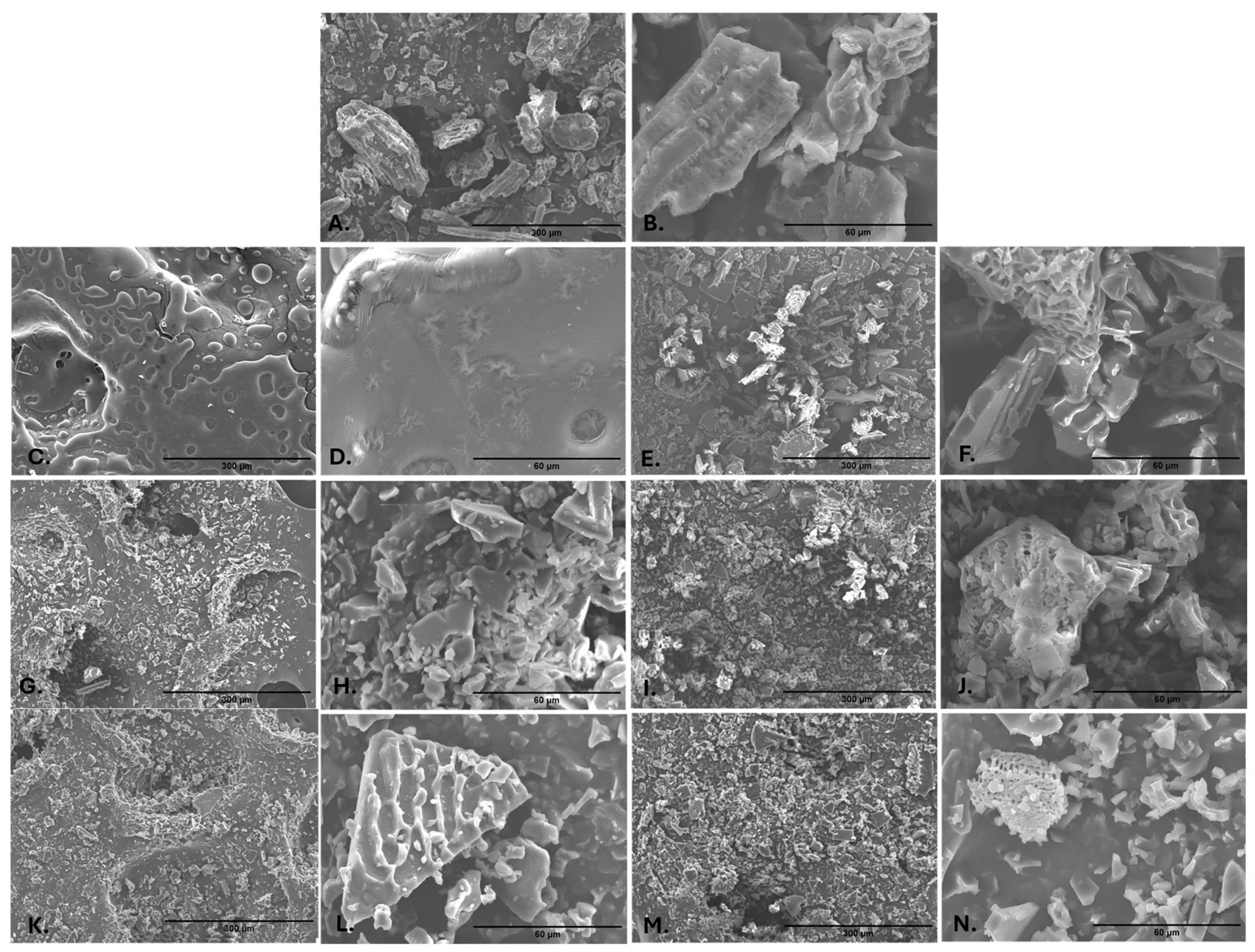
Scanning electron microscopy (SEM) images of coffee pulp, microencapsulation formulations, and their respective controls at 200× and 1000×, obtained at an accelerating voltage of 20 kV. (**A**) ES (200×); (**B**) ES (1000×); (**C**) EMD (200×); (**D**) EMD (1000×); (**E**) AMD (200×); (**F**) AMD (1000×); (**G**) EPR (200×); (**H**) EPR (1000×); (**I**) APR (200×); (**J**) APR (1000×); (**K**) EMP (200×); (**L**) EMP (1000×); (**M**) AMP (200×); (**N**) AMP (1000×). Scale bars: 300 µm (200×) and 60 µm (1000×).

The morphology of ES and lyophilized formulations was evaluated by scanning electron microscopy (SEM) and is presented in Figure 1. ES (Figure 1A) showed particles with irregular morphology and high size heterogeneity, presenting both very small and larger particles, with a porous surface and no defined shape. In contrast, all lyophilized formulations (EMD, EPR, and EMP) and their respective controls (AMD, APR, and AMP) showed a lamellar morphology with flake-like structures of different sizes.

#### 2.2.3. FT-IR Spectroscopy

**Figure 2:**
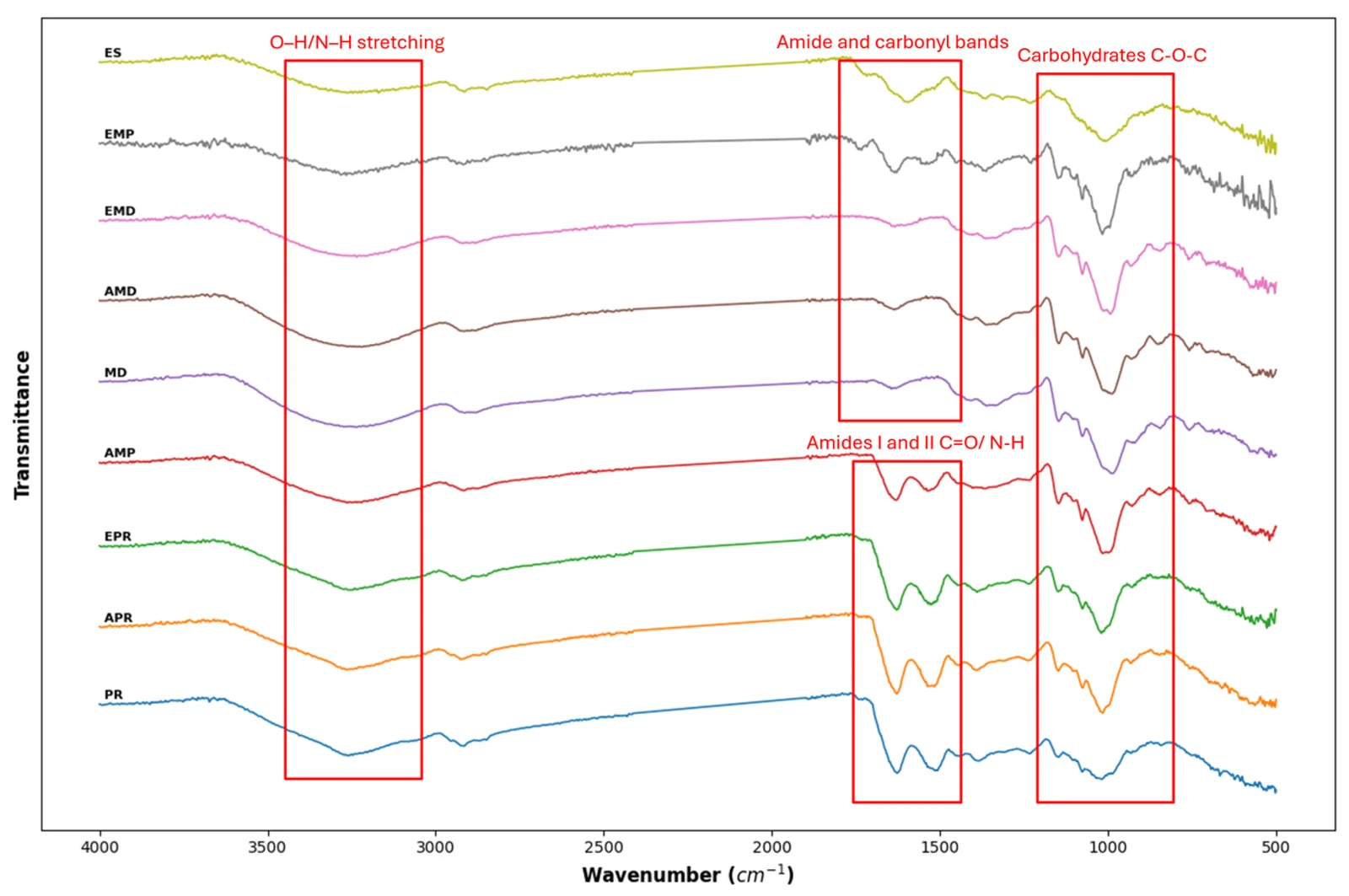
FT-IR spectra of dried coffee pulp (ES), microencapsulation formulations (EMD, EPR, EMP), control formulations (AMD, APR, AMP), and pure wall materials (maltodextrin, MD; whey protein isolate, PR), recorded by ATR from 4000 to 500 cm^−1^. Spectra are vertically offset for clarity; boxes indicate the main regions discussed in the text.

The interaction between wall materials and the core was analyzed by FT-IR spectroscopy. Formulations (EMD, EPR, and EMP), their respective controls (AMD, APR, and AMP), pure wall materials (MD and PR), and coffee pulp (ES) were analyzed; their FT-IR spectra are presented in Figure 2. In the 3000–3500 cm^−1^ region, a broad O-H/N-H stretching band was identified in all formulations, and was more pronounced in whey protein-containing samples (PR, APR, and EPR) and attenuated in ES and EMP. In the 1536–1629 cm^−1^ region, Amide I and II bands (C=O and N-H), characteristic of whey protein isolate, were identified with high intensity in PR, APR, and EPR, and remained well defined in AMP, whereas in EMP they appeared attenuated and less defined. In ES, a band at 1594 cm^−1^ corresponding to C=C and C=O vibrations was observed. In the 900–1200 cm^−1^ region, C-O-C stretching bands characteristic of maltodextrin were observed in MD, AMD, and EMD, with a defined peak at 986 cm^−1^.

#### 2.2.4. Thermogravimetric Analysis (TGA/DTG)

**Figure 3:**
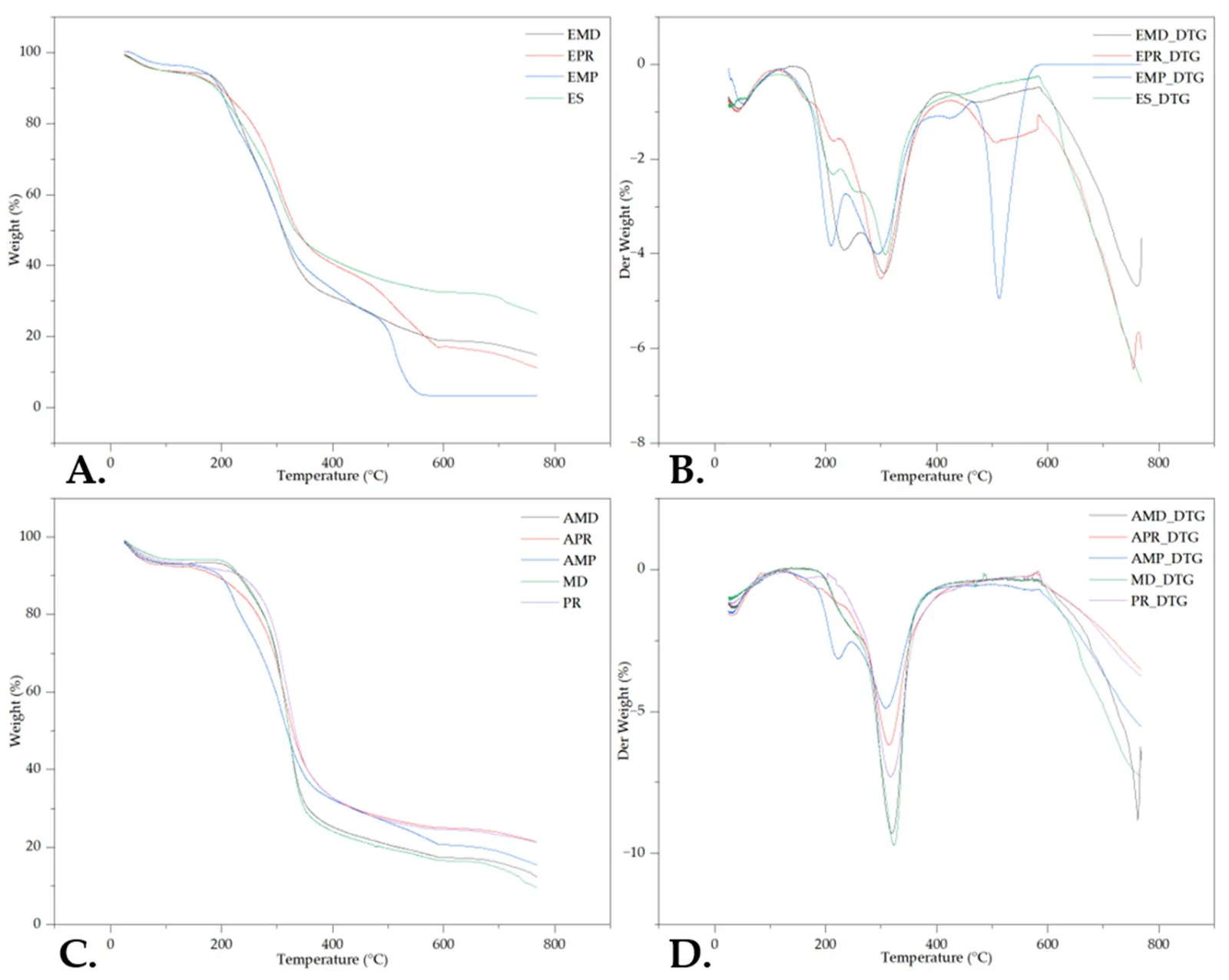
Thermogravimetric analysis (TGA) and derivative thermogravimetric analysis (DTG) of microencapsulation formulations and pure wall materials. (**A**) TGA curves of extract-containing formulations (EMD, EPR, EMP) and dried coffee pulp (ES); (**B**) DTG curves of extract-containing formulations and ES; (**C**) TGA curves of control formulations (AMD, APR, AMP) and pure wall materials (MD, PR); (**D**) DTG curves of control formulations and pure wall materials.

The thermal behavior of ES, pure wall materials, and lyophilized formulations was evaluated by TGA and DTG and is presented in Figure 3. All samples showed three degradation stages. In the first stage (25–200 °C), mass loss associated with the evaporation of free and bound water was observed, with values ranging from 5.13% (MD) to 11.39% (EMP). In the second stage (200–400 °C), the greatest mass loss occurred in all formulations, with values between 46.50% (ES) and 69.81% (MD). The main DTG peaks were located between 294 °C (EMP) and 323 °C (MD). ES, EMD, EPR and EMP, as well as AMP, showed an additional DTG peak or shoulder between 209–232 °C, absent in AMD, APR, MD, and PR. In the third stage (above 400 °C), EMP showed a pronounced degradation event at 513 °C, and EPR showed a smaller event in the same region, whereas no comparable event was observed in the controls without extract or in the pure wall materials. Accordingly, mass loss in the third stage was higher in EMP (30.26%) and EPR (29.19%) than in the other samples (11.42–16.84%). ES showed the highest final residue (26.61%), while EMP showed the lowest (3.45%).

### 2.3. Total Phenolic and Flavonoid Contents

**Figure 4:**
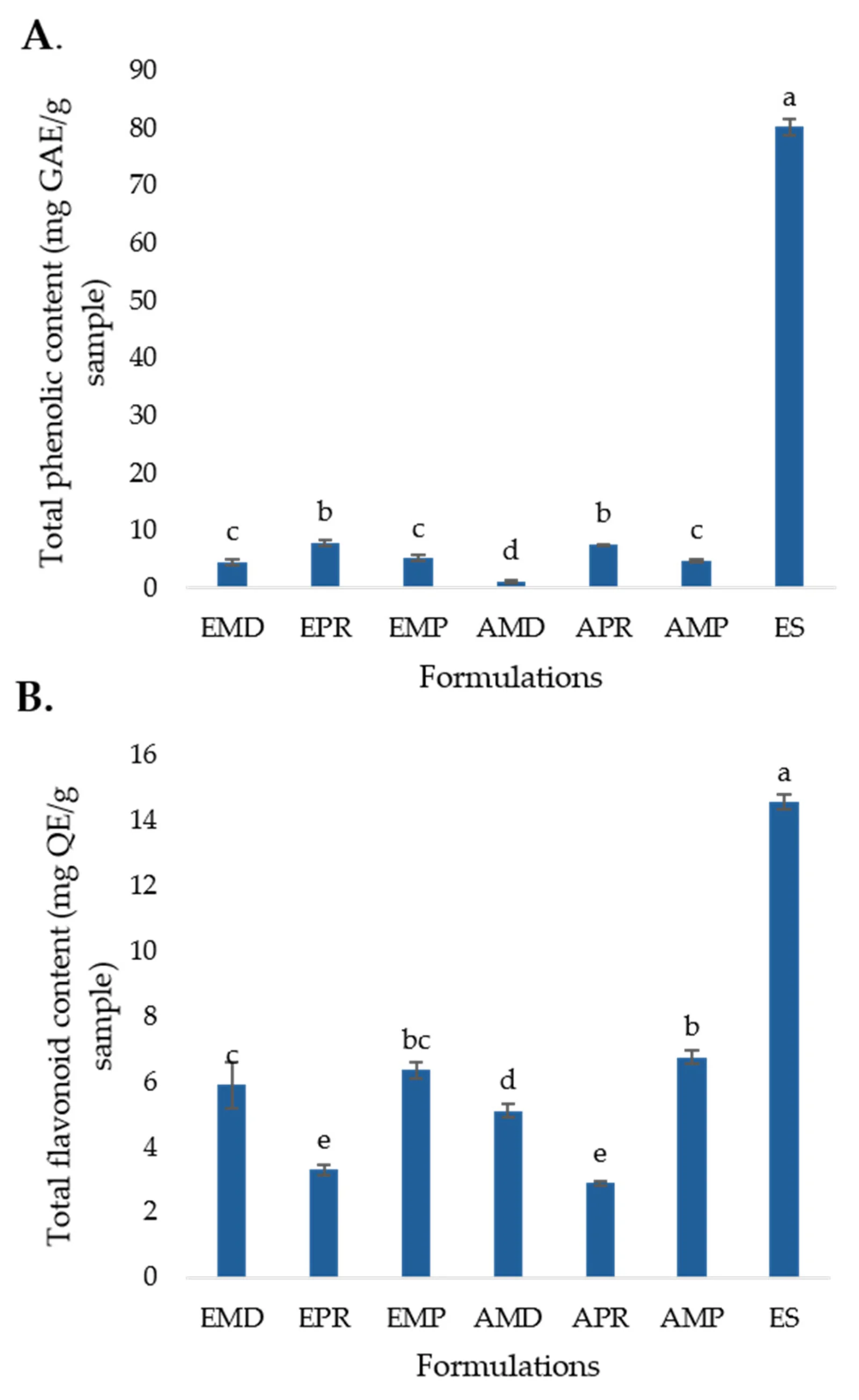
Total phenolic content (**A**) and total flavonoid content (**B**) of the dried coffee pulp (ES) and microencapsulation formulations. Bars represent mean ± standard deviation (*n* = 3). Different letters above bars indicate statistically significant differences among formulations (*p* < 0.05, Tukey’s test). GAE: gallic acid equivalents; QE: quercetin equivalents.

Total phenolic (TPC) and flavonoid (TFC) contents of all formulations and the dried coffee pulp are presented in Figure 4. ES showed the highest TPC (80.23 ± 1.49 mg GAE/g sample), which was significantly higher than that of all formulations (*p* < 0.05). Among encapsulated formulations, EPR showed the highest TPC (7.82 ± 0.50 mg GAE/g sample), followed by EMP (5.13 ± 0.49 mg GAE/g sample) and EMD (4.45 ± 0.45 mg GAE/g sample), with no statistically significant differences between EMD and EMP. Control formulations showed similar behavior: APR (7.56 ± 0.07 mg GAE/g sample) was statistically equal to EPR, AMP (4.64 ± 0.21 mg GAE/g sample) was statistically equal to EMD and EMP, and AMD showed the lowest value among all formulations (1.02 ± 0.15 mg GAE/g sample).

Regarding TFC, ES again showed the highest value (14.55 ± 0.22 mg QE/g sample). Among extract-containing formulations, EMP showed the highest content (6.34 ± 0.26 mg QE/g sample), followed by EMD (5.89 ± 0.70 mg QE/g sample), with no statistically significant differences between them, while EPR showed the lowest value (3.29 ± 0.16 mg QE/g sample). Among controls, AMP showed the highest TFC (6.74 ± 0.22 mg QE/g sample), followed by AMD (5.10 ± 0.21 mg QE/g sample) and APR (2.89 ± 0.08 mg QE/g sample). When each formulation was compared with its respective control, only EMD differed statistically in both TPC and TFC, whereas EPR and EMP showed values statistically equal to those of APR and AMP, respectively, reflecting the response of both assays to the wall materials, particularly to whey protein.

### 2.4. Encapsulation Efficiency (EE)

**Figure 5:**
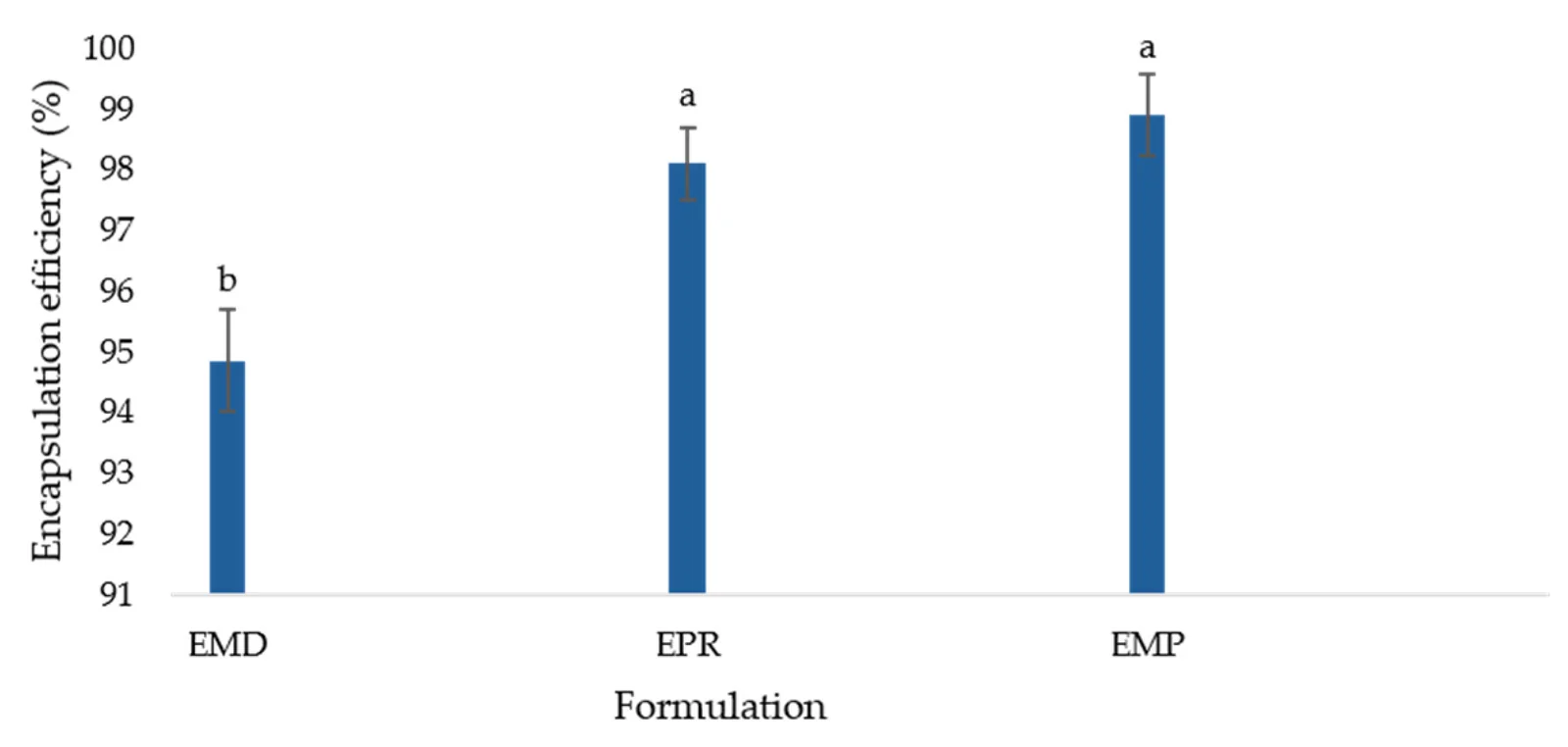
Encapsulation efficiency (EE%) of microencapsulation formulations. Bars represent mean ± standard deviation (*n* = 3). Different letters above bars indicate statistically significant differences among formulations (*p* < 0.05, Tukey’s test).

The encapsulation efficiency (EE) of the three formulations is presented in Figure 5. All formulations showed EE values above 94%. EPR and EMP showed the highest values, with 98.10 ± 0.59% and 98.90 ± 0.66%, respectively, with no statistically significant differences between them. EMD showed the lowest value (94.86 ± 0.83%), which was statistically different from EPR and EMP.

### 2.5. Antioxidant Capacity

**Figure 6:**
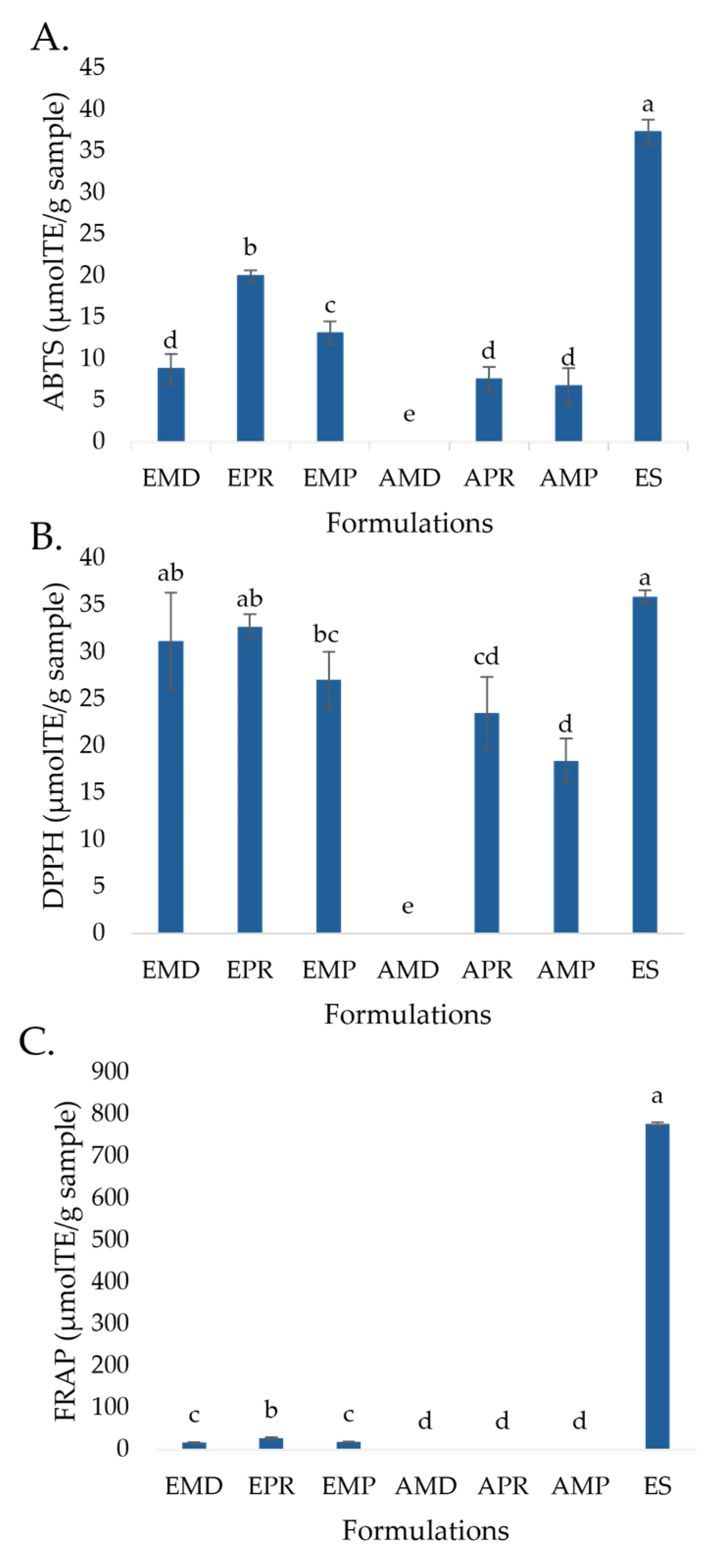
Antioxidant capacity evaluated by ABTS (**A**), DPPH (**B**), and FRAP (**C**) assays for the dried coffee pulp (ES) and microencapsulation formulations. Bars represent mean ± standard deviation (*n* = 3). Different letters above bars indicate statistically significant differences among formulations (*p* < 0.05, Tukey’s test). Values for AMD in ABTS and DPPH, and for AMD, APR, and AMP in FRAP, correspond to non-detectable values under the assay conditions.

The antioxidant capacity of all formulations and the dried coffee pulp, evaluated by ABTS, DPPH, and FRAP assays, is presented in Figure 6. In all three assays, ES showed the highest antioxidant capacity, with values of 37.34 ± 1.48 µmol TE/g sample (ABTS), 35.87 ± 0.69 µmol TE/g sample (DPPH), and 778.16 ± 2.89 µmol TE/g sample (FRAP), which were statistically different from all other formulations in the ABTS and FRAP assays (*p* < 0.05). AMD showed no detectable antioxidant capacity in any of the three assays, and APR and AMP also showed no detectable activity in FRAP.

In the ABTS assay, among extract-containing formulations, EPR showed the highest value (19.97 ± 0.69 µmol TE/g sample), followed by EMP (13.12 ± 1.38 µmol TE/g sample) and EMD (8.79 ± 1.80 µmol TE/g sample), all statistically different from each other. APR (7.56 ± 1.43 µmol TE/g sample) and AMP (6.74 ± 2.12 µmol TE/g sample) showed no statistically significant differences between them or relative to EMD.

In the DPPH assay, EMD (31.20 ± 5.10 µmol TE/g sample) and EPR (32.69 ± 1.34 µmol TE/g sample) showed no statistically significant differences between them or relative to ES, while EMP (27.04 ± 2.97 µmol TE/g sample) showed a significantly lower value. Among controls, APR (23.49 ± 3.84 µmol TE/g sample) showed higher activity than AMP (18.42 ± 2.35 µmol TE/g sample).

In the FRAP assay, differences among formulations were more pronounced. EPR showed the highest value among encapsulated formulations (27.23 ± 2.17 µmol TE/g sample), followed by EMP (18.50 ± 1.72 µmol TE/g sample) and EMD (17.14 ± 1.52 µmol TE/g sample), with no statistically significant differences between EMD and EMP. Control formulations AMD, APR, and AMP showed no detectable antioxidant capacity by this method.

### 2.6. Antimicrobial Activity (MIC and MBC)

MIC and MBC values of the dried coffee pulp and microencapsulation formulations against *E. coli* and *S. aureus* are presented in Table 2. All evaluated formulations showed inhibitory and bactericidal activity against both strains within the evaluated concentration range (1–10 mg/mL).

Against *E. coli*, EMD and EPR showed the lowest MICs (≤1 and 1 mg/mL, respectively), while EMP and ES showed MICs of 2 and 4 mg/mL, respectively. Regarding MBC against *E. coli*, EMD showed the lowest value (1 mg/mL), followed by EPR (2 mg/mL), while EMP and ES required concentrations of 6 mg/mL to achieve a bactericidal effect.

For *S. aureus*, EMD, EPR, and EMP showed the same MIC (1 mg/mL), outperforming ES, which required 2 mg/mL. In MBC against *S. aureus*, EMD and EPR showed an MBC of 2 mg/mL, ES required 6 mg/mL, and EMP needed the highest concentration evaluated (10 mg/mL) to achieve complete eradication.

## 3. Discussion

Lyophilization yield is an indicator of wall material efficiency and process effectiveness, with direct implications for the cost of wall materials and active compounds used [29]. The yields obtained for extract-containing formulations (78.99–81.49%) were slightly lower than those reported by Azarpazhooh et al. [30] for a plant extract freeze-dried with maltodextrin, gum arabic, and whey protein isolate (84.06–89.45%). This difference may be related to the calculation basis, since those authors expressed the yield relative to the dry weight of the wall materials, whereas in the present study the extract solids were also included, as well as to the powder losses that occurred during grinding with a coffee grinder and sieving through a 0.300 mm mesh, which is finer than the 500 µm mesh used by those authors. EMD and EMP showed the highest yields (81.17% and 81.49%, respectively). In the case of EMD, this behavior can be attributed to the inherent properties of maltodextrin, including its high solubility, low viscosity, and ability to form a protective layer around particles during processing, reducing adhesion in the lyophilizer chamber and thereby improving powder recovery; in fact, Azarpazhooh et al. [30] also obtained the highest yield with maltodextrin alone (89.45%). EPR showed a lower yield (78.99%), which agrees with the lower yield reported by those authors for whey protein isolate alone (86.86%) and can be explained by the foaming capacity of whey protein isolate [31], which can hinder complete powder recovery during processing. The yield of EMP (81.49%) was similar to that of EMD, which contrasts with findings reported by Azarpazhooh et al. [30], who found that a binary combination of maltodextrin and whey protein isolate at a 50:50 ratio resulted in a lower yield (85.91%) than either material used alone. This result suggests that under the conditions employed in the present study, maltodextrin in the mixture exerted a stabilizing effect on the foam generated by the protein, which is consistent with the reported use of maltodextrin as a stabilizer of foamed structures in freeze-drying processes [32], thereby counteracting the yield loss associated with EPR.

Water activity (a_w_) reflects the equilibrium moisture of a material and represents the water available to interact with the environment, including microorganisms whose growth is favored at elevated a_w_ levels [33]. In this study, all a_w_ values obtained for encapsulated formulations (0.321–0.467) were below the threshold of 0.6 reported as the limit for microbiological or biochemical stability [34]. These low values agree with those reported by Araújo et al. [25] for spent coffee ground extracts encapsulated with different wall materials, which were particularly low in freeze-dried powders. Although these values are favorable, they do not by themselves establish the stability of the microparticles during storage, which should be confirmed in further studies. ES showed an a_w_ value of 0.498 ± 0.005, close to that reported by Barrios-Rodríguez et al. [35] for Castillo cultivar coffee pulp (0.34), and comparable to values found in other plant-based powder matrices such as apple pulp (0.431) [36].

Moisture content is a key factor determining the shelf life of powdered products, as high values can promote particle agglomeration and microbial growth, with consequent physical, chemical, and sensory changes [25]. ES showed a moisture content of 9.97 ± 0.03%, which is higher than that reported by Barrios-Rodríguez et al. [35] for Castillo cultivar coffee pulp (6.1%), a difference that can be attributed to the different drying temperatures employed, with the temperature used in this study being 15 °C lower than that reported by those authors. In another study on forced convection drying of coffee pulp using the same temperature as the present study (50 °C), a moisture content of 13.93% was reported [37], higher than that obtained here, a difference that could be attributed to differences in particle size before moisture measurement. Encapsulated formulations showed moisture contents between 1.88% and 4.40%, comparable to or lower than those reported in the literature for freeze-dried microcapsules (3.12–7.05%) [38]. EMP showed the lowest moisture content (1.88 ± 0.04%), which could be attributed to the combination of maltodextrin and whey protein isolate generating a matrix with lower water retention capacity compared to maltodextrin alone. EMD showed the highest moisture content among encapsulated formulations (4.40 ± 0.20%), consistent with findings reported in the literature, where a higher proportion of maltodextrin is associated with higher moisture content, as its numerous hydroxyl (-OH) groups form hydrogen bonds with water molecules, favoring retention [39]. Differences in moisture content among whey protein-containing formulations may be related to the differential affinity of proteins for water and their different water diffusion coefficients through the wall material [23].

Color is an important property in microencapsulated products, as it allows quality evaluation and describes the final appearance of the product; moreover, it is a selection criterion for consumers that determines product acceptance or rejection [34,40]. ES showed a low L* value, indicating a dark powder, while positive a*, b*, and C* values reflect a tendency toward reddish and yellowish hues, consistent with findings reported in the literature for processed coffee pulp, where powders tend to show positive a* values and low L*, reflecting the presence of reddish and dark pigments characteristic of this by-product (Table 1) [35].

Following encapsulation with maltodextrin and whey protein isolate, powder lightness increased significantly compared to ES, while maintaining a tendency toward reddish and yellowish hues. Control formulations showed the highest L* values and a more pronounced tendency toward yellow-green, essentially reflecting the color of the wall materials in the absence of extract. The difference observed in the b* parameter between extract-containing formulations and their respective controls suggests that an interaction between the extract and the wall material occurs during the encapsulation process. Likewise, the high lightness of encapsulated formulations may be attributed to the wall materials, which visually attenuated the original color of the pulp [39,40].

Microscopic observation of coffee pulp and lyophilized formulations (Figure 1) yielded results consistent with those reported in the literature for freeze-dried powders, where lamellar structures of different sizes are described as a common characteristic of lyophilized products [23]. This morphology can be attributed to the low temperatures involved in freeze-drying, which result in insufficient forces to break the frozen liquid into droplets, generating flatter and more irregular structures [41]. Other authors have reported similar findings and described microcapsules with deformed, irregular, and flaky structures [42,43,44]. However, since SEM only shows the external morphology of the powders, these observations do not allow a core–shell structure or the location of the extract within the particles to be confirmed.

Fourier-transform infrared (FT-IR) spectroscopy is a versatile analytical tool with a wide range of applications, including the structural analysis of molecules and their polymers, allowing the identification of chemical bonds characteristic of each substance [33]. The spectrum recorded for ES (Figure 2) showed an O-H/N-H stretching band at 3247 cm^−1^, a region characteristic of hydroxyl groups present in phenolic compounds such as chlorogenic acids or tannins, and structural polymers such as cellulose and hemicellulose of coffee pulp [45,46]. Additionally, a band at 1594 cm^−1^ was identified in ES, corresponding to C=C and C=O vibrations associated with aromatic compounds and phenolic esters in the pulp, which may be associated with lignin, polyphenols, chlorogenic acids, and caffeine [45,46]. Finally, a band at 1007 cm^−1^ was found, corresponding to C-O and C-O-C bonds associated with cellulose and hemicellulose [46].

In maltodextrin-containing formulations (EMD and AMD), characteristic carbohydrate bands were observed in the 900–1200 cm^−1^ region, with a defined peak at 986 cm^−1^ associated with C-O-C stretching characteristic of maltodextrin. Changes in this region have been used in the literature to determine modifications in polysaccharide structure within microcapsules [22]. In whey protein-containing formulations (EPR and APR), Amide I and Amide II regions, characteristic of pure whey protein isolate (PR), were clearly identified, consistent with findings reported in the literature, where these regions are described as key indicators of protein structure in microcapsules [22].

The most relevant observation was in EMP, where the Amide I and Amide II bands appeared attenuated and less defined compared with AMP, which contains the same proportion of whey protein isolate without extract. The O-H/N-H stretching band around 3262 cm^−1^ was also attenuated. Zhao et al. [23] reported that flavonoids can interact with whey protein through electrostatic forces, as evidenced by shifts in the amide region and the disappearance of N-H bending bands in protein core mixtures. However, in microcapsules combining whey protein isolate and maltodextrin, the hydroxyl band shift was slighter than in protein core mixtures, which the authors attributed to the incorporation of maltodextrin. Accordingly, the changes observed in EMP may suggest interactions between the extract compounds and the wall materials, possibly modulated by the presence of maltodextrin, although FT-IR alone does not allow these interactions to be identified specifically.

Thermogravimetric evaluation revealed mass loss in the first stage (25–200 °C), corresponding to the evaporation of free and bound water through hydrogen bonds, behavior widely reported in the literature [24,47]. The second degradation stage, with DTG peaks between 294 and 323 °C, shows differentiated behavior according to the wall material: in maltodextrin-containing formulations it corresponds to the rupture of glycosidic bonds and decomposition of carbohydrate polymers, including carbohydrate ring dehydration and depolymerization [48], while in whey protein-containing formulations it is associated with protein denaturation and decomposition, for which peaks between 250 and 334 °C have been reported depending on the protein proportion in the formulation [49,50]. The DTG peak of ES at 307 °C is consistent with findings reported by Zuluaga et al. [46], who described a prominent peak at 303 °C corresponding to the degradation of galactomannans, cellulose, and arabinogalactans in coffee grounds.

A DTG peak or shoulder between 209 and 232 °C was observed in ES and in the extract-containing formulations, as well as in AMP, but not in AMD, APR, MD, or PR, suggesting that in the extract-containing samples it may be associated with the degradation of extract compounds. The third degradation event was most pronounced in EMP at 513 °C, while EPR showed a smaller event in the same region and no comparable event was observed in the controls without extract or in the pure wall materials. This distinctive thermal feature, together with the attenuation of the Amide I and II bands observed by FT-IR, could be related to interactions among maltodextrin, whey protein, and the polyphenols of the extract, since protein–polysaccharide–polyphenol ternary complexes have been reported to show thermal and functional properties that differ from those of their individual components [51]. However, this event involved additional mass loss, and EMP showed the lowest final residue (3.45%), so it does not indicate greater thermal stability. Moreover, FT-IR and TGA/DTG provide only indirect evidence of such interactions; confirming the formation of a ternary complex would require more direct techniques, such as fluorescence spectroscopy or X-ray diffraction, which were not included in this study. Finally, the higher final residue of ES (26.61%) compared to encapsulated formulations may be attributed to the higher content of inorganic compounds and minerals present in coffee pulp [46].

Total phenolic and flavonoid content analyses are important tools in the pharmaceutical, cosmetic, and food industries, used to ensure the quality of functional foods and evaluate the efficacy of antioxidants in the prevention of diseases associated with oxidative stress [33]. ES showed the highest total phenolic content (80.23 ± 1.49 mg GAE/g sample), a value higher than that reported for Catimor cultivar coffee pulp dried under similar conditions (11.34 mg GAE/g) [45] and close to that reported by Barbosa et al. [26] for coffee silverskin extract (74.78 mg GAE/g), which confirms that coffee by-products are rich in phenolic compounds.

Following microencapsulation, all extract-containing formulations showed a significant reduction in total phenolic content compared to ES. A similar behavior was reported by Silva et al. [15] for coffee husk extract encapsulated with maltodextrin and whey protein concentrate, whose phenolic content decreased from 761.22 mg CAE/g in the free extract to 28.29–29.38 mg CAE/g in the microparticles, a difference that the authors attributed mainly to the dilution effect of the wall materials. In the present study, this dilution effect is also expected, since the extract represented only a small fraction of the solids of each formulation. Likewise, part of the phenolic compounds may remain bound to the wall materials, particularly to whey protein, and may not be fully released during sample preparation for the assay, which would lead to an underestimation of their content in the formulations. Although EPR showed the highest TPC among the formulations, this result should be interpreted with caution, since APR showed a statistically equal value. As explained by Pérez et al. [52], the Folin–Ciocalteu reagent is not specific to phenolic compounds and also reacts with other reducing substances, such as aromatic amino acids present in proteins. For this reason, encapsulation efficiency was calculated using extract-free blanks to correct this interference (Section 4.6.3).

As with phenolics, all encapsulated formulations showed lower TFC than ES, and EPR showed the lowest value among them (Figure 4). This behavior contrasts with that observed for total phenolics, and part of this difference can be explained by the response of each assay. While the Folin–Ciocalteu reagent also responds to the protein, as discussed above, the AlCl_3_ method depends on the formation of complexes between aluminum and specific groups of the flavonoid structure, and Shraim et al. [53] showed that its response varies considerably depending on the flavonoids present and the standard used. In this regard, Tazeddinova et al. [54] describe that whey protein binds polyphenols through hydrogen bonds with their hydroxyl groups; thus, if part of the flavonoids remains bound to the protein in EPR, fewer groups would be available to form complexes with aluminum, which could explain its lower TFC. This is supported by the fact that EPR and EMP showed TFC values statistically equal to those of their respective controls, indicating that the wall materials contributed substantially to the response of this assay.

Encapsulation efficiency (EE) measures the capacity of the wall material to retain compounds. The evaluated formulations showed EE values above 94%, indicating high retention of phenolic compounds. EMD showed the lowest EE (94.86 ± 0.83%), although this value falls within the range reported by da Silva Júnior et al. [42] for ciriguela peel extract encapsulated with maltodextrin alone (76.83–98.23%). The values of EPR (98.10 ± 0.59%) and EMP (98.90 ± 0.66%) are comparable to those obtained by Zhao et al. [23] for *Cornus officinalis* flavonoids encapsulated with whey protein isolate and maltodextrin (93.29–98.24%), who observed that a lower proportion of protein was less favorable for the formation of the protective film of the microcapsules. These results are also consistent with findings reported by Barbosa et al. [26], who reported that whey protein concentrate and skimmed milk powder preserved the phenolic content and antioxidant activity of coffee silverskin extract [26]. Likewise, Vázquez-Núñez et al. [27] reported an EE of 98% for coffee extract encapsulated in whey protein isolate, a value very close to that obtained for EPR (98.10%) and EMP (98.90%) in the present study.

The quantification of antioxidant capacity of natural compounds allows characterization and evaluation of their use in combating oxidative stress [55]. Different techniques have been developed to evaluate this potential in plant-based products; however, antioxidant capacity depends on the compounds’ ability to donate hydrogen, electrons, or chelate metals [55,56]. ES showed the highest antioxidant capacity in the three assays (Figure 6), and its ABTS (37.34 µmol TE/g) and DPPH (35.87 µmol TE/g) values are close to those reported by Rendón-Mera et al. [45] for Catimor coffee pulp oven-dried at the same temperature used in this study (50 °C), with ABTS values of 51.68–64.55 µmol TE/g and DPPH values of 31.43–62.97 µmol TE/g; the authors also observed that oven drying reduced antioxidant capacity compared with freeze-drying, due to prolonged thermal exposure. This behavior indicates that ES is neutralizing the radicals through the two main pathways: electron and hydrogen atom transfer for ABTS and DPPH, and electron transfer for FRAP [56].

After microencapsulation, all formulations showed lower antioxidant capacity than ES, especially in the ABTS and FRAP assays. As discussed for phenolic content, this reduction can be mainly attributed to the dilution of the extract by the wall materials, as reported by Silva et al. [15], and possibly to the binding of part of the phenolic compounds to the wall materials, particularly to whey protein, which may limit their availability to react [54]. In the DPPH assay, EMD and EPR showed no statistically significant differences compared to ES; since antioxidant assays differ in their reaction mechanisms and conditions [56], the response of the encapsulated compounds may vary depending on the assay used. In the ABTS and FRAP assays, EPR showed the highest antioxidant capacity among formulations, which agrees with the influence of the wall material observed by Silva et al. [15] in coffee husk microparticles, where formulations combining maltodextrin and whey protein concentrate showed the highest ABTS (up to 76.9% inhibition) and FRAP values, and whey protein-containing formulations even increased their antioxidant capacity after intestinal digestion, which the authors attributed to the release of protected polyphenols and the generation of antioxidant peptides from protein hydrolysis. This behavior can be explained by the fact that whey protein isolate contains amino acids with intrinsic antioxidant capacity, such as cysteine with free thiol groups and tryptophan, while maltodextrin acts as a physical barrier without contributing antioxidant capacity [19,57]. This is consistent with findings reported by Zhao et al. [23], who found that the combination of maltodextrin and whey protein isolate was the formulation with the highest antioxidant capacity when encapsulating *Cornus officinalis* extract.

The MIC corresponds to the lowest concentration of an antimicrobial capable of inhibiting visible growth of a microorganism [58]. ES showed an MIC of 4 mg/mL against *E. coli* and 2 mg/mL against *S. aureus*, indicating a greater inhibitory effect on Gram-positive bacteria. This is consistent with findings reported in the literature, where coffee pulp and silverskin extracts require lower concentrations to inhibit the growth of Gram-positive bacteria [59], a pattern also observed by Duangjai et al. [10] for coffee pulp extract. Regarding the magnitude of these values, Maia et al. [60], in their review of the antimicrobial properties of coffee by-products, compiled MIC values of 4.69 mg/mL for coffee pulp extract and 5 mg/mL for spent coffee grounds extract against Gram-positive bacteria, and noted that coffee pulp extract showed no bactericidal effect even at 300 mg/mL. The MIC of ES (2–4 mg/mL, expressed as dried pulp powder) is within the same order of magnitude as these values, whereas the encapsulated formulations showed lower MIC (≤1–2 mg/mL) and MBC values (1–10 mg/mL), which places them in a favorable position compared with previously reported coffee by-product extracts. Moreover, to our knowledge, the effect of different wall materials on the antimicrobial activity of encapsulated coffee by-product extracts had not been previously compared.

Compared with ES, all encapsulated formulations showed lower MIC values against both strains, and EMD and EPR also showed lower MBC values. This could be related to the fact that the compounds of ES were not protected by a wall material and were directly exposed to environmental conditions during the incubation, which could have promoted their oxidation and reduced their availability to interact with bacteria. As pointed out by do Carmo et al. [11], the compounds present in free extracts are susceptible to oxidation and degradation when exposed to environmental factors such as heat and light, which would reduce their availability to interact with bacteria. Among the encapsulated formulations, EMD showed the lowest MBC against *E. coli*, and EMD and EPR showed the same MIC and MBC against *S. aureus*. A similar behavior was reported by Ferreira-Santos et al. [61], who observed that pine bark extract encapsulated with maltodextrin was effective against bacteria and attributed this effect to the wall material, which would improve the solubility of the extract compounds and facilitate their entry into bacterial cells. Once released, these compounds could act through the mechanisms described by Pinto et al. [62], who explain that chlorogenic acids and tannins reduce extracellular pH and alter cell membranes, inhibiting oxidative phosphorylation and depriving microorganisms of the nutrients necessary for growth. Regarding *E. coli,* although Gram-negative bacteria are generally less susceptible because their lipopolysaccharide-rich outer membrane limits the entry of antibacterial compounds, as observed for ES, Castro-Díaz et al. [63] describe in their review studies in which coffee extracts disrupted this outer membrane, and Chen et al. [64] showed that chlorogenic acid, the main phenolic compound in coffee pulp, damaged the cell membrane and increased the permeability of a Gram-negative bacterium. Thus, a greater availability of these compounds in EMD may have been sufficient to overcome this barrier. On the other hand, the lower activity of EPR and EMP could be related to the binding of part of the phenolic compounds to whey protein; in this regard, Nassarawa et al. [65] point out that the tendency of polyphenols to form complexes with proteins can neutralize their antibacterial potential.

Since none of the formulations was superior in all the properties evaluated (for instance, EPR showed the lowest flavonoid content and EMD the highest moisture content among the encapsulated formulations), the choice of wall material should depend on the intended application. The results obtained in this study also open the way for complementary analyses. Spectrophotometric methods allowed the total phenolic and flavonoid contents of the formulations to be determined and compared; however, a chromatographic characterization (e.g., HPLC) of the extract and the microparticles is recommended to identify the specific compounds, such as the chlorogenic acids, 5-caffeoylquinic acid, and gallic acid reported in coffee pulp, responsible for the biological activities observed. To broaden the scope of this work, it would also be advisable to evaluate the stability of the microparticles during storage, monitoring their phenolic content, color, and antioxidant capacity over time, as well as their behavior under simulated gastrointestinal digestion, since this information would provide a complete picture to support their use as functional food ingredients.

## 4. Materials and Methods

### 4.1. Coffee Pulp Collection and Preparation

Coffee pulp was obtained from Castillo^®^ cultivar coffee cherries harvested at the Buenavista farm, located in Vereda La Topacia Alta, municipality of Génova, southern Quindío department, Colombia, at an altitude of 1801 m a.s.l. (4°17′02.4″N, 75°46′05.8″W). A total of 5110 g of fresh pulp was collected and dried by forced convection at 50 °C for 24 h, yielding 790 g of dry material. The dried pulp was ground using a coffee grinder (GX4100, KRUPS, Offenbach am Main, Germany) and sieved through a 0.300 mm mesh (FTL-0200, Iris, Badalona, Spain).

### 4.2. Chemicals and Reagents

Folin–Ciocalteu reagent, sodium carbonate (Na_2_CO_3_), aluminum chloride (AlCl_3_), potassium persulfate, sodium acetate trihydrate, ferric chloride hexahydrate (FeCl_3_·6H_2_O), 2,4,6-tris(2-pyridyl)-s-triazine (TPTZ), 2,2-diphenyl-1-picrylhydrazyl (DPPH), 2,2′-azino-bis (3-ethylbenzothiazoline-6-sulfonic acid) (ABTS), and Trolox standard were purchased from Sigma-Aldrich (St. Louis, MO, USA). Maltodextrin was purchased from McKenna Group (Bogotá, Colombia). Whey protein isolate was purchased from Natulifestyle (Bogotá, Colombia). Luria–Bertani (LB) broth was purchased from Condalab (Madrid, Spain) and Mueller-Hinton agar (MHA) from Bioxon (Mexico City, Mexico).

### 4.3. Preparation of Aqueous Extract

The aqueous extract was prepared by mixing 1 g of dried and sieved pulp with 10 mL of filtered water (1:10 *w*/*v*). The mixture was subsequently subjected to ultrasonic bath treatment for 15 min (Zokop, Experty, Fuzhou, China), centrifuged at 5000 rpm for 15 min (Z 206 A, Hermle, Gosheim, Germany), and the resulting supernatant was collected and reserved for subsequent analyses.

### 4.4. Preparation of Freeze-Dried Microcapsules

For microcapsule preparation, the aqueous coffee pulp extract was mixed with wall materials, which were incorporated at a concentration of 20% (*w*/*v*), a concentration previously used in the freeze-drying encapsulation of spent coffee grounds extracts [25]. Three wall material formulations were evaluated: maltodextrin alone, whey protein isolate alone, and a 1:1 combination of both (50/50), a proportion previously evaluated in protein-maltodextrin wall systems for freeze-dried microcapsules [30]. As a control, wall materials were dissolved in distilled water following the same procedure. Mixtures were stirred on a magnetic stir plate until complete homogenization was achieved.

Subsequently, the mixtures were frozen at −20 °C for 5 days and freeze-dried using a lyophilizer (FDU-2100, Eyela, Tokyo, Japan) at −80 °C and 10.6 Pa for 24 h. The lyophilized material was ground using a coffee grinder (GX4100, KRUPS, Offenbach am Main, Germany), sieved through a 0.300 mm mesh using a sieve shaker (FTL-0200, Iris, Baladona, Spain), and stored in a silica gel desiccator at room temperature until use.

Lyophilization yield was calculated according to Equation (1), where *m_powder* corresponds to the mass of powder recovered after lyophilization and grinding; *m_WM* corresponds to the mass of wall material added; and *m_ES* corresponds to the soluble solids contributed by the extract, estimated from its volume and soluble solids content (2.7°Brix). For control formulations, *m_ES* was zero.

(1) $$Yield\;(\%)=\left(\frac{m\_powder}{m\_WM+m\_ES}\right)\times 100$$

### 4.5. Characterization of Microparticles

#### 4.5.1. Color

To evaluate the color contribution of the extract to each wall material and the appearance differences among the obtained powders, color was measured using a spectrophotocolorimeter (NS800, Shenzhen 3NH Technology, Shenzhen, China) based on CIELab color space parameters. The total color difference (∆E) was calculated according to Equation (2):

(2) $$∆E=\sqrt{{{(∆L}^{*})}^{2}}+{{(∆a}^{*})}^{2}+{{(∆b}^{*})}^{2}$$

where ∆L*, ∆a*, and ∆b* represent the differences in lightness, red–green chromaticity, and yellow–blue chromaticity, respectively, between each sample and the dried coffee pulp (ES), which was used as the reference.

#### 4.5.2. Water Activity and Moisture Content

Water activity was measured in triplicate using a water activity meter (AquaLab Series 3, METER Group, Inc. formerly Decagon Devices, Pullman, WA, USA). Moisture content was determined gravimetrically using a universal drying oven (UF55, Memmert, Schwabach, Germany) at 105 °C [33].

#### 4.5.3. Scanning Electron Microscopy (SEM)

The surface morphology of lyophilized powders was evaluated by scanning electron microscopy (SEM). Samples were adhered to an aluminum pin stub using conductive carbon tape and coated with a thin gold layer. Micrographs were obtained at an accelerating voltage of 20 kV using a scanning electron microscope (JSM-7800F, JEOL, Tokyo, Japan).

#### 4.5.4. Fourier-Transform Infrared (FT-IR) Spectroscopy

Interactions between wall materials and compounds present in the extract were analyzed using an FT-IR spectrometer (Spectrum GX, PerkinElmer, Waltham, MA, USA) equipped with an attenuated total reflectance (ATR) accessory over a spectral range of 4000 to 500 cm^−1^. Measurements were performed in transmittance mode, and all samples were analyzed in triplicate.

#### 4.5.5. Thermogravimetric Analysis (TGA)

The thermal properties of the samples were determined using a thermogravimetric analyzer (Pyris 1, PerkinElmer, Waltham, MA, USA) under an inert nitrogen atmosphere at a flow rate of 20 mL/min. Samples were heated from 25 to 800 °C at a constant heating rate of 10 °C/min.

### 4.6. Total Phenolic, Flavonoid Contents, and Antioxidant Capacity

#### 4.6.1. Sample Preparation

For the determination of total phenolic and flavonoid contents and antioxidant capacity, lyophilized powders and dried and sieved pulp were prepared by dissolving 0.05 g of each sample in 5 mL of 40% (*v*/*v*) methanol. Mixtures were subjected to ultrasonic bath treatment for 15 min at room temperature (Zokop, Experty, Fuzhou, China) and centrifuged at 5000 rpm for 15 min (Z 206 A, Hermle, Germany). The resulting supernatant was collected and reserved for subsequent analyses. All results were expressed per gram of sample, corresponding to the total powder for the formulations, including the wall materials, and to the dried pulp for ES.

#### 4.6.2. Total Phenolic and Flavonoid Contents

Total phenolic and flavonoid contents were determined following the microplate-adapted methodology described by Garzón-García et al. [66]. Total phenolic content (TPC) was determined using the Folin–Ciocalteu method. In each microwell, 10 µL of microparticle suspension and 25 µL of 1 N Folin–Ciocalteu reagent were added and allowed to rest for 5 min under refrigeration. Subsequently, 25 µL of 20% Na_2_CO_3_ and 140 µL of distilled water were added, and the mixture was kept at rest for 30 min at room temperature in the dark. Absorbance was measured at 760 nm using a microplate reader (Multiskan GO, Thermo Fisher Scientific, Waltham, MA, USA). The calibration curve was constructed with gallic acid over a concentration range of 0.01 to 0.6 mg/mL (R^2^ = 0.997), and results were expressed as mg of gallic acid equivalents per gram of lyophilized material (mg GAE/g).

Total flavonoid content (TFC) was determined by mixing 80 µL of microparticle suspension with 80 µL of ethanolic aluminum chloride solution. The mixture was agitated for 30 s and left to rest in the dark for 1 h at room temperature, after which it was agitated again for 30 s prior to measuring absorbance at 400 nm using the same microplate reader. The calibration curve was constructed with quercetin over a concentration range of 0.01 to 0.6 mg/mL (R^2^ = 0.994), and results were expressed as mg of quercetin equivalents per gram sample (mg QE/g).

#### 4.6.3. Encapsulation Efficiency (EE)

Encapsulation efficiency (EE) was determined following the methodology described by Estrella-Osuna et al. [67], based on the total phenolic content measured as described in Section 4.6.2. Spectrophotometric interference caused by the reaction of the Folin–Ciocalteu reagent with the wall materials was corrected by subtracting the values of extract-free controls processed under the same conditions. EE was calculated according to Equation (3):

(3) $$EE\%=\left(\frac{C_{1}-C_{2}}{C_{1}}\right)\times 100$$

where *C*_1_ is the initial encapsulated concentration and *C*_2_ is the final encapsulated concentration.

#### 4.6.4. Antioxidant Capacity Assays

Antioxidant capacity was evaluated using three assays: 2,2-diphenyl-1-picrylhydrazyl radical scavenging (DPPH), 2,2′-azino-bis(3-ethylbenzothiazoline-6-sulfonic acid) (ABTS), and ferric reducing antioxidant power (FRAP), following the methodology described by Pérez-Pérez et al. [68] with some modifications.

For the DPPH assay, 1.25 mg of DPPH radical (Sigma-Aldrich) was dissolved in 50 mL of pure methanol and the absorbance was adjusted to 0.7 at 515 nm. In each well, 20 µL of microparticle suspension and 220 µL of DPPH solution were added, incubated for 30 min in the dark, and absorbance was measured at 515 nm using the microplate reader. The calibration curve was constructed with Trolox standard (Sigma-Aldrich) over a range of 0 to 0.1 mg/mL (R^2^ = 0.985), and results were expressed as µmol Trolox equivalents per gram of sample (µmol TE/g).

For the ABTS assay, two solutions were prepared: 19.5 mg of ABTS (Sigma-Aldrich) dissolved in 5 mL of distilled water, and 37.8 mg of potassium persulfate dissolved in 1 mL of distilled water. Eighty-eight microliters of the persulfate solution were mixed with the ABTS solution and left to rest in the dark for 24 h. Subsequently, 200 µL of this mixture was diluted in 20 mL of ethanol and the absorbance was adjusted to 0.7 at 734 nm. In each well, 20 µL of microparticle suspension and 270 µL of the adjusted ABTS radical solution were added, incubated for 30 min in the dark at room temperature, and absorbance was measured at 734 nm. The calibration curve was constructed with Trolox standard (Sigma-Aldrich) over a range of 0 to 0.1 mg/mL (R^2^ = 0.994), and results were expressed as µmol Trolox equivalents per gram of sample (µmol TE/g).

For the FRAP assay, three stock solutions were prepared: sodium acetate trihydrate buffer (1143 mg in 22 mL of distilled water, pH 3.6), 2,4,6-tris(2-pyridyl)-s-triazine (TPTZ; Sigma-Aldrich) solution (31.23 mg in 10 mL of 40 mM HCl), and FeCl_3_·6H_2_O solution (15.1 mg in 2.8 mL of distilled water). The working solution was prepared by mixing these three stock solutions in a buffer:FeCl_3_·6H_2_O:TPTZ ratio of 10:1:1 (*v*/*v*/*v*). In each well, 20 µL of microparticle suspension and 280 µL of working solution were added, incubated for 30 min in the dark at room temperature, and absorbance was measured at 638 nm. The calibration curve was constructed with Trolox standard (Sigma-Aldrich) over a range of 0 to 0.8 mg/mL (R^2^ = 0.992), and results were expressed as µmol Trolox equivalents per gram of sample (µmol TE/g).

### 4.7. Determination of Minimum Inhibitory Concentration (MIC) and Minimum Bactericidal Concentration (MBC).

The antimicrobial activity of the dried coffee pulp (ES) and lyophilized powders was evaluated against two reference strains: *Escherichia coli* (ATCC 23216) and *Staphylococcus aureus* (ATCC 25923). Both strains were reactivated in Luria–Bertani (LB) broth at 37 °C for 16 h, then streaked onto Mueller-Hinton agar (MHA) plates and incubated again at 37 °C for 16 h. Inocula were prepared at a concentration of 10^4^ CFU/mL in LB broth.

Stock solutions of each sample (10 mg/mL, expressed as mg of powder per mL) were diluted in LB broth to obtain concentrations of 8, 6, 4, 2, and 1 mg/mL. In each microplate well, in triplicate, 295 µL of sample solution and 5 µL of 10^4^ CFU/mL inoculum were added (final inoculum of approximately 1.7 × 10^2^ CFU/mL). LB broth with inoculum, LB broth without inoculum, and sample solutions without inoculum were included as growth, sterility, and blank controls, respectively. Microplates were incubated at 37 °C for 17 h, after which 20 µL from each well were plated on MHA and incubated at 37 °C for 17 h. The MIC was defined as the lowest concentration at which growth was reduced compared with the growth control, and the MBC as the lowest concentration at which no growth was observed. Wall-material controls (AMD, APR, and AMP) were evaluated in a preliminary assay and showed no inhibition of either strain; therefore, MIC and MBC were determined only for ES and the extract-containing formulations.

### 4.8. Statistical Analysis

All experiments were performed in triplicate (*n* = 3) and results were expressed as mean ± standard deviation. Data normality was assessed using the Shapiro–Wilk test and homogeneity of variances using Bartlett’s test. One-way analysis of variance (ANOVA) followed by Tukey’s multiple comparison test (*p* < 0.05) was applied to identify significant differences among treatments. Statistical analyses were performed using R (version 4.5.2; R Foundation for Statistical Computing, Vienna, Austria) in RStudio (Posit Software, Boston, MA, USA) and Microsoft Excel (Microsoft Corporation, Redmond, WA, USA). Thermogravimetric profiles (TGA/DTG) and FT-IR spectra were processed and plotted using OriginPro (version 10.1.0.178, OriginLab Corporation, Northampton, MA, USA).

## 5. Conclusions

The present study evaluated the microencapsulation by freeze-drying of aqueous coffee pulp extract from the Castillo^®^ cultivar using maltodextrin (EMD), whey protein isolate (EPR), and a 1:1 combination of both (EMP) as wall materials, characterizing the obtained microparticles in terms of physicochemical properties, surface morphology, molecular interactions, thermal behavior, encapsulation efficiency, total phenolic and flavonoid contents, antioxidant capacity, and antimicrobial activity against *Escherichia coli* and *Staphylococcus aureus*. The results showed that all formulations exceeded 94% encapsulation efficiency, with EPR and EMP reaching values close to 99%. Each wall material conferred different advantages: EPR showed the highest antioxidant capacity among the formulations in the ABTS and FRAP assays, EMD showed the lowest MBC against *E. coli* and, together with EPR, the lowest MIC and MBC values against *S. aureus* and EMP showed the lowest moisture content and water activity. Additionally, EMP showed a distinctive thermal degradation event at 513 °C and attenuated Amide I and II bands in FT-IR, which may indicate interactions between the wall materials and the extract compounds. Overall, these findings support the valorization of coffee pulp, an underutilized agro-industrial by-product, and show that freeze-dried microparticles of its aqueous extract exhibit antioxidant and antimicrobial activity, with properties that depend on the wall material used.

## Figures and Tables

**Table 1:** Moisture content, water activity, and CIELab color parameters of microencapsulation formulations and dried coffee pulp.

Formulations	Moisture (%)	a_w_	Color Analysis					
			L*	a*	b*	∆E	C*	h°
ES	9.97 ± 0.03 a	0.498 ± 0.005 b	40.189 ± 0.002 g	13.646 ± 0.004 a	26.000 ± 0.009 a	-	29.363 ± 0.006 a	62.307 ± 0.014 g
EMD	4.40 ± 0.20 bc	0.467 ± 0.005 c	84.206 ± 0.003 d	3.407 ± 0.002 d	17.140 ± 0.003 c	46.052 ± 0.001 d	17.475 ± 0.003 c	78.756 ± 0.006 d
EPR	3.62 ± 0.25 c	0.453 ± 0.020 c	83.003 ± 0.001 f	3.530 ± 0.001 b	17.522 ± 0.001 b	44.802 ± 0.000 f	17.874 ± 0.001 b	78.610 ± 0.003 e
EMP	1.88 ± 0.04 d	0.321 ± 0.003 d	83.175 ± 0.001 e	3.424 ± 0.001 c	16.567 ± 0.001 d	45.180 ± 0.000 e	16.917 ± 0.001 d	78.323 ± 0.003 f
AMD	4.26 ± 0.34 bc	0.569 ± 0.004 a	94.633 ± 0.005 a	−0.042 ± 0.001 e	2.943 ± 0.002 g	60.688 ± 0.003 a	2.943 ± 0.002 g	90.814 ± 0.024 c
APR	4.58 ± 0.64 b	0.473 ± 0.004 c	92.729 ± 0.001 c	−0.312 ± 0.002 g	8.688 ± 0.002 e	57.052 ± 0.000 c	8.694 ± 0.002 e	92.055 ± 0.011 a
AMP	4.56 ± 0.11 b	0.517 ± 0.003 b	94.203 ± 0.000 b	−0.165 ± 0.001 f	6.457 ± 0.002 f	59.077 ± 0.000 b	6.459 ± 0.002 f	91.464 ± 0.010 b

ES: dried coffee pulp; EMD: extract encapsulated with maltodextrin; EPR: extract encapsulated with whey protein isolate; EMP: extract encapsulated with 1:1 maltodextrin/whey protein isolate combination; AMD: maltodextrin control; APR: whey protein isolate control; AMP: 1:1 combination control. L*: lightness; a*: red–green chromaticity; b*: yellow–blue chromaticity; ∆E: total color difference relative to ES; C*: chroma; h°: hue angle. Different letters within the same column indicate statistically significant differences among formulations (*p* < 0.05, Tukey’s test).

**Table 2:** Minimum inhibitory concentration (MIC) and minimum bactericidal concentration (MBC) of the dried coffee pulp (ES) and microencapsulation formulations against *Escherichia coli* and *Staphylococcus aureus*.

	*Escherichia coli*		*Staphylococcus aureus*	
Formulation	MIC (mg/mL)	MBC (mg/mL)	MIC (mg/mL)	MBC (mg/mL)
EMD	≤1	1	1	2
EPR	1	2	1	2
EMP	2	6	1	10
ES	4	6	2	6

MIC: minimum inhibitory concentration; MBC: minimum bactericidal concentration. Concentrations evaluated were 1, 2, 4, 6, 8, and 10 mg/mL. ≤1: inhibition observed at the lowest concentration evaluated.

## Data Availability

The authors are available to share raw data upon request.
